# Unlocking the depths: multiple factors contribute to risk for hypoxic blackout during deep freediving

**DOI:** 10.1007/s00421-023-05250-z

**Published:** 2023-06-10

**Authors:** Eric Mulder, Craig Staunton, Arne Sieber, Erika Schagatay

**Affiliations:** 1 Environmental Physiology Group, Department of Health Sciences, Mid Sweden University, Kunskapens Väg 8, 831 25 Östersund, Sweden; 2 Swedish Winter Sports Research Centre, Mid Sweden University, Östersund, Sweden; 3 Oxygen Scientific GmbH, Graz, Austria

**Keywords:** Apneic diving, Breath-hold diving, Diving response, Syncope, Shallow water blackout, Bradycardia

## Abstract

**Purpose:**

To examine the effect of freediving depth on risk for hypoxic blackout by recording arterial oxygen saturation (SpO_2_) and heart rate (HR) during deep and shallow dives in the sea.

**Methods:**

Fourteen competitive freedivers conducted open-water training dives wearing a water-/pressure proof pulse oximeter continuously recording HR and SpO_2_. Dives were divided into deep (> 35 m) and shallow (10–25 m) *post-hoc* and data from one deep and one shallow dive from 10 divers were compared.

**Results:**

Mean ± SD depth was 53 ± 14 m for deep and 17 ± 4 m for shallow dives. Respective dive durations (120 ± 18 s and 116 ± 43 s) did not differ. Deep dives resulted in lower minimum SpO_2_ (58 ± 17%) compared with shallow dives (74 ± 17%; *P* = 0.029). Overall diving HR was 7 bpm higher in deep dives (*P* = 0.002) although minimum HR was similar in both types of dives (39 bpm). Three divers desaturated early at depth, of which two exhibited severe hypoxia (SpO_2_ ≤ 65%) upon resurfacing. Additionally, four divers developed severe hypoxia after dives.

**Conclusions:**

Despite similar dive durations, oxygen desaturation was greater during deep dives, confirming increased risk of hypoxic blackout with increased depth. In addition to the rapid drop in alveolar pressure and oxygen uptake during ascent, several other risk factors associated with deep freediving were identified, including higher swimming effort and oxygen consumption, a compromised diving response, an autonomic conflict possibly causing arrhythmias, and compromised oxygen uptake at depth by lung compression possibly leading to atelectasis or pulmonary edema in some individuals. Individuals with elevated risk could likely be identified using wearable technology.

## Introduction

Freediving, or breath-hold diving, is both a recreational- and a competitive sport. The competitive context is also known as apnea, in which athletes compete in various disciplines after a single breath of air. These include static apnea, where one maintains the longest possible apneic duration at the water surface (Schagatay 2009); dynamic apnea, where one swims the longest possible distance underwater in a shallow pool; (Schagatay 2010) and constant weight and free immersion, in which one descends to the greatest possible depth using different methods (Schagatay 2011). The swimming disciplines include categories with or without fins of different types.

As all disciplines of competitive apnea are performed on a single inhalation of air, arterial oxygen saturation will progressively decrease during the apnea as internal oxygen stores are consumed (Andersson and Schagatay 1998a). Enhanced tolerance to asphyxia is, therefore, necessary to sustain long apneic events (Schagatay 2011). Conservation of oxygen is facilitated by the human cardiovascular “diving response” (Andersson et al. 2002; Alboni et al. 2011), whereby parasympathetically elicited bradycardia and sympathetically activated peripheral vasoconstriction directs blood flow to vital organs (Foster and Sheel 2005). Work economy also influences oxygen stores in the disciplines requiring movement, as the diving metabolic rate and heart rate (HR) are affected by the level of exertion (Mulder et al. 2021a). Other apnea-prolonging features include large lung and spleen volumes (Schagatay et al. 2012), the latter of which contracts during apnea, thereby increasing circulating red cell volume and subsequent oxygen availability (Schagatay et al. 2001; Schagatay et al. 2012).

Prolonged dives will nonetheless lead to decreased arterial oxygen saturation, eventually impairing normal brain function, which may result in hypoxic syncope, also called blackout (Craig 1961). Without assistance, drowning will likely occur. In competitive situations, this risk is limited by safety divers who assist in resurfacing and resumed breathing (Schagatay 2011).

Freediving to depth may increase the risk of blackout due to the decreasing hydrostatic pressure while ascending to the surface, causing “shallow water blackout” (Lanphier and Rahn 1963). Here, oxygen exchange may cease or even be reversed, moving oxygen from the blood back into the lungs (Lanphier and Rahn 1963). Study of such real-world situations has historically been restricted due to limitations in measurement techniques.

Deep freediving may also compromise lung function through atelectasis, pulmonary oedema, and hemoptysis (Linér and Andersson 2008; Mijacika and Dujic 2016; Schipke et al. 2019; Barković et al. 2021; Valdivia-Valdivia et al. 2021). These conditions can hamper alveolar oxygen extraction during the remaining dive, and delayed recovery of arterial oxygen saturation measured by pulse oximetry (SpO_2_) is often seen after deep dives and increases with depth (Linér and Andersson 2008). Recent studies of blood gases during freediving in a deep diving pool showed that some divers developed hypoxia at depth, despite the expected hyperoxia (Bosco et al. 2018). The authors ascribed the hypoxia in these individuals to atelectasis, leading to a ventilation/perfusion mismatch and a greater physiological shunt. There is also evidence that barotrauma may be more common than reported. Patrician et al. (2021) recently confirmed by ultrasound that remaining low SpO_2_ after deep dives reflected lung fluid accumulation.

A well-developed cardiovascular diving response may thus protect the diver against negative effects of depth. Lemaître et al. (2013) found in an EKG study of deep dives that the bradycardia stimulus dominated over the exercise tachycardia. It has, however, not been clarified if the diving response develops differently with depth. Several characteristics of deep diving could conceivably affect the response. Lung compression at depth reduces lung volume, which laboratory-based studies have shown to enhance diving response (Andersson and Schagatay 1998b), as does lower water temperature (Schuitema and Holm 1988; Schagatay and Holm 1996). Differentiating the influence of these potentially multiple factors requires studying HR and SpO_2_ during real dives of varying depths. Wearable measurement devices can make this possible. In a pilot study using a novel underwater pulse oximeter (Mulder et al. 2021b), a rapid decrease in SpO_2_ during the ascent phase from freedives to depth was observed (Mulder et al. 2021a). The oxygen desaturation was less pronounced in shorter, shallow dives by the same individuals, but whether this was due to pressure change, apnea duration or other factors is unclear.

The aim of the current study was, therefore, to differentiate the contributions of multiple factors to oxygen desaturation when diving to different depths during dives of similar duration, using continuous SpO_2_ and HR measurement via underwater pulse oximetry. We hypothesized that deeper dives would result in lower oxygen saturation levels during ascent compared to shallow dives, while other variables would not differ.

## Methods

### Participants

Ten male and four female healthy, elite competitive freedivers (mean ± SD age 38 ± 9 years, height 178 ± 9 cm and body mass 71 ± 9 kg) volunteered for the study after on-site convenience recruitment. All participants had a minimum of five years of freediving experience and were currently conducting regular training for, and participating in, major freediving competitions. All performed at a level equivalent to category 4 or 5 of the 5-level categorization system for freediving training/experience (Andersson and Schagatay 1998b). Participants’ mean (±  SD) personal best competition performance in any deep diving discipline was 72 ± 19 m. All participants were provided verbal information about the study and consented in writing before inclusion in the study. The study protocol was approved by the Regional Committee for Medical and Health Research Ethics in Umeå, Sweden, and tests were conducted in accordance with the 2013 Declaration of Helsinki with the exemption of research pre-registration in a publicly accessible database.

### Equipment

A fit-for-purpose submersible pulse oximeter, consisting of two identical reflective sensor heads which were placed on the temples and connected by cables to a data logger, was used to record SpO_2_ and HR during dives. The model of the prototype used was previously described and evaluated in detail (Mulder et al. 2021b); it is based on a prototype previously employed to shallow depths (Kuch et al. 2010; Kiviniemi et al. 2012). Prior to the dives, the sensors’ placement and function was confirmed through visual inspection of the live plethysmograph signals on a laptop. Both sensors monitored SpO_2_ and HR continuously across the pre-dive preparations, dives, and recovery periods. The data logger, connected to the sensors, was then placed inside the wetsuit on the back of the freediver to minimize potential disruption. The logger also monitored temperature, depth, and time.

All divers wore a long sleeve wetsuit with a neoprene thickness of three or five millimeters, according to their own preference. They used the facial equipment of their choice; this included a diving mask covering the eyes and nose, glasses filled with water in combination with a nose clip, or only a nose clip. All divers used their own diving watch to monitor dive time and depth.

### Procedures

All measurements were performed in the morning in accordance with the divers’ planned training, in a voluntary fasted state since the previous evening. Divers were then instructed about how recordings would be conducted and how they should proceed with their dives, specifically including the following:

- To execute their dive routines as they had planned in terms of number of dives, dive depths, and/or time between dives, without guidance from the research team
- To use the same dive technique regardless of depth, if possible
- To avoid touching the equipment sensors, if possible
- That a researcher would observe the divers in-water in a non-intrusive manner.

Following the instructions, divers were allowed to undertake their individual pre-dive routines, involving breathing- and stretching exercises, meditation, and/or yoga. When the divers indicated that they were ready to dive, they were equipped with the underwater pulse oximeter. They then entered the water, accompanied by at least one experienced safety diver of their own choice. All dives were performed from a freediving buoy with a weighted vertical line set at a self- and pre-determined depth. The diver was attached to the vertical line by a safety lanyard which prevented descent beyond the pre-determined depth but allowed early return if desired. All measurements were performed in open water, with mean water surface temperature of 22 °C and an ambient air temperature of 26 °C. Water temperature was decreasing with depth.

**Fig. 1:**
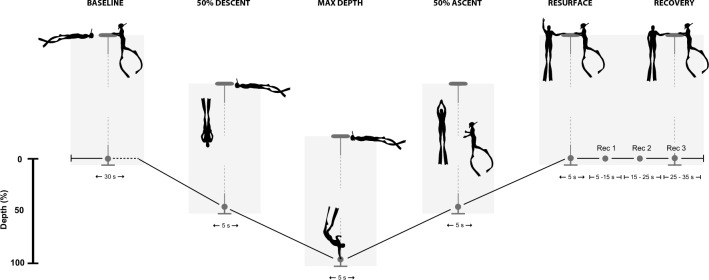
Schematic representation of different dive points used for the analysis. Calculations of each point consists of the mean of a 10 s window surrounding the following points concerning dive depth: the halfway point during descent (50% descent), maximum depth (max depth), halfway point during ascent (50% ascent) and upon resurfacing (resurface). Recovery following resurfacing was analyzed in three consecutive 10 s windows beginning 5 s after resurfacing (rec 1, 2 and 3). Baseline was calculated as the mean of a 60 s window preceding the onset of the dive

None of the freedivers were observed hyperventilating prior to diving, but all used the “lung packing” method to increase lung volume above total lung capacity (Örnhagen et al. 1998). The divers then dove towards their planned depths using constant weight (with or without fins) or by free immersion. At the bottom plate, divers turned and started their ascent. Divers were accompanied by their safety diver during the last 20 m (± 5 m) of ascent (Fig. 1). Upon resurfacing, all divers performed “hook breathing” (Fernandez et al. 2019) followed by the surface protocol employed in freediving competitions confirming a conscious and aware state (Schagatay 2011). Each diver conducted all dives during one training session on the same day, with most performing several dives to different depths with a self-determined period of rest between each. Data logging was ended, and all equipment removed when the divers indicated they had completed their training session.

### Inclusion and exclusion criteria

**Fig. 2:**
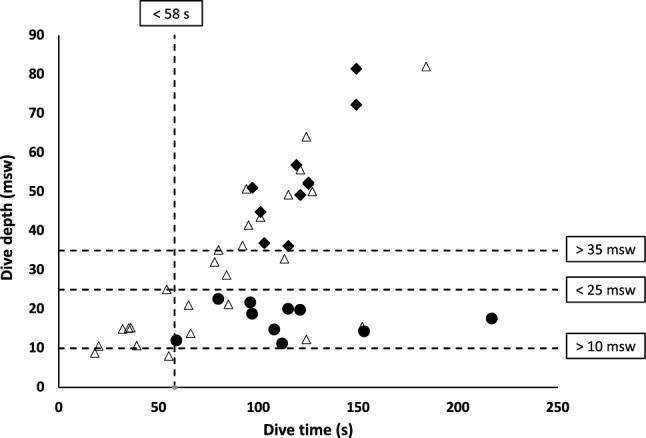
Dive depth and duration of the included deep (> 35 m, closed diamonds) and shallow (10–25 m, closed circles) dives for the 10 participants. Open triangles represent excluded dives according to exclusion criteria and method analysis description

A total of 46 spontaneous dives were recorded from 14 divers. Depths attained during diving were classified *post-hoc* as deep (> 35 msw) or shallow (10–25 msw), while depths attained between 25 and 35 msw were excluded from analysis (Fig. 2). One deep and one shallow dive in the same discipline was included per diver, unless any of the following exclusion criteria were met: dive duration < 1 min; pre-dive recovery interval < 3 min; or insufficient photoplethysmogram quality. In four divers, all conducted dives were excluded for one or more of these reasons. The 10 remaining divers produced four dives on average (range 2–5) of which six dives were excluded according to the exclusion criteria, and 29 dives were included for potential analysis. If several dives per diver met the inclusion criteria, the deepest dive of the deep dives and the longest duration dive of the shallow dives were included in the analysis (Fig. 2). On average, the included shallow dive was the second dive performed (range 1–3), and the included deep dive was the third dive performed (range 1–4) in the individuals’ series. The included deep dives corresponded to ~ 75% of the divers’ personal best.

### Data analysis

**Fig. 3:**
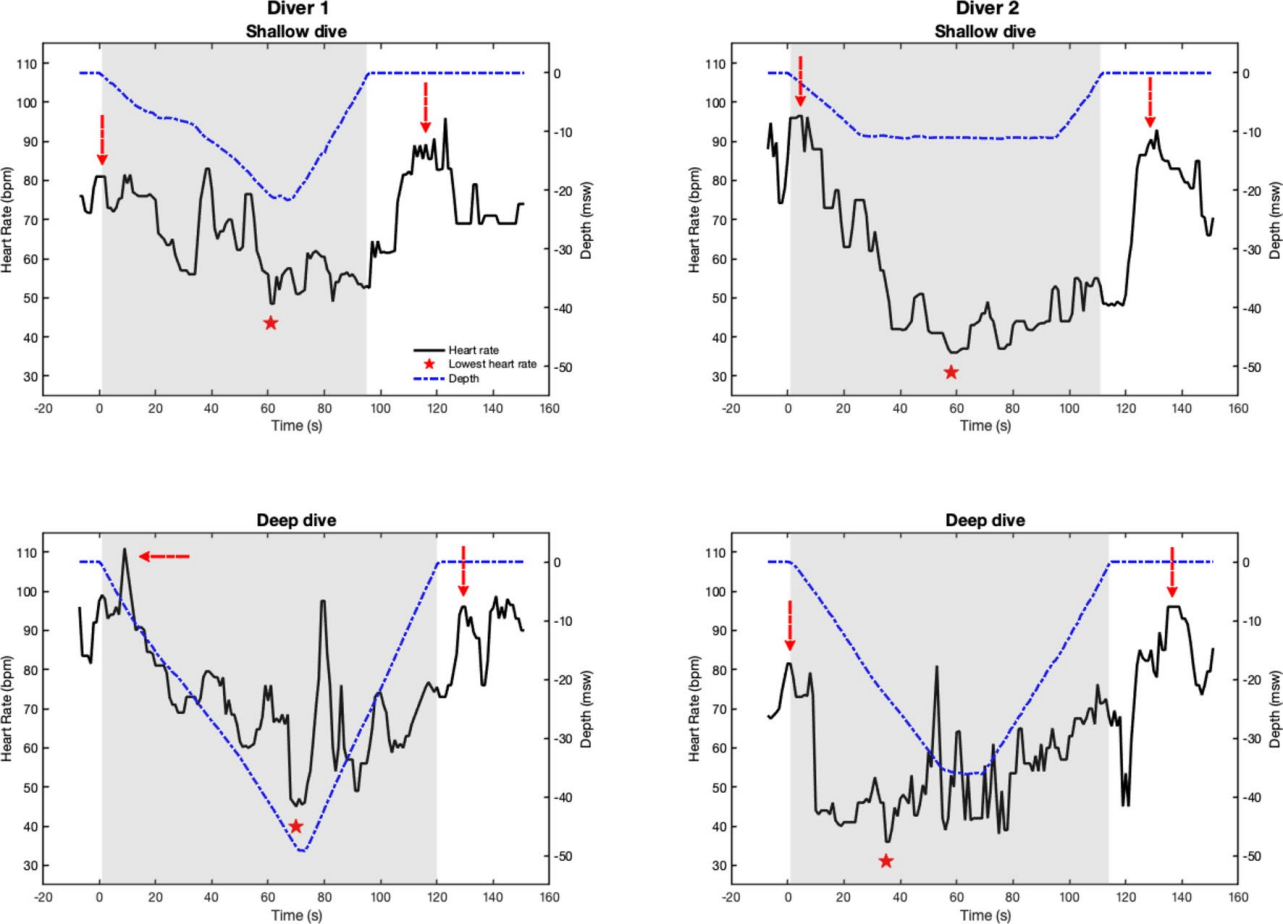
Individual recordings for two divers performing one shallow and one deep dive each, with data plotted second by second. Black line represents heart rate, blue dash-dotted line represents depth. Shaded area indicates the dive period. Red stars represent the HR_min_ for each dive. Red arrows indicate tachycardia at start and end of dives

Second-by-second measurements of the two simultaneously recording sensors were averaged to obtain one single value of SpO_2_ and HR per second. To avoid including outlier values caused by artefacts, data were visually examined and smoothed using a 5 s moving median function. The resultant HR patterns from two divers are shown in Fig. 3. Baseline SpO_2_ and HR were determined for each dive separately by calculating the mean of the 90 to 30 s period preceding the onset of the dive, which has been shown to be the most stable (Andersson and Schagatay 1998a; Mulder et al. 2021a). The lowest attained SpO_2_ (SpO_2min_) from the start of one dive until one minute after resurfacing was noted. The diving HR was calculated as the mean HR recorded from the start to the end of the dive. The lowest HR during the dive (HR_min_), and the corresponding time point for this, were also noted.

To allow comparison of dives of varying durations between individuals, mean SpO_2_ and HR were calculated for 10 s periods surrounding the following points concerning dive depth: the halfway point during descent (50% descent), maximum depth (max depth), halfway point during ascent (50% ascent) and upon resurfacing (resurface). Recovery following resurfacing was analyzed in three consecutive 10 s windows beginning 5 s after resurfacing (Fig. 1).

### Statistical analysis

A Shapiro–Wilk test was used to check the normalcy of distribution of the data; no deviations from normal distribution were found. Group data were expressed as mean ± SD. Two-way repeated measures ANOVAs (within factors: Depth and Dive Point) were used to determine the effect of diving depth (Shallow OR Deep) on the pattern of SpO_2_ and HR response throughout the dive. Greenhouse–Geisser corrections were applied if the assumption of sphericity could not be made. Significant interactions, identified at *α* ≤ 0.05, were followed up with simple main effect analyses with pairwise comparisons using Bonferroni correction. Paired sample t-tests were also used to assess differences (identified at *α* ≤ 0.05) in dive duration, SpO_2min_, diving HR, HR reduction, HR_min_, timepoint for HR_min_ and temperature between shallow and deep dives. Pearson correlation was used to examine relationships between SpO_2_ at Max Depth and upon Resurface, as well as for temperature drop and diving depth, and r values reported. Statistical analyses were performed using IBM SPSS Statistics (Version 27.0; IBM Corporation, NY).

## Results

The mean ± SD attained depth was 17 ± 4 msw for the shallow dives, and 53 ± 14 msw for the deep dives. The mean duration of the shallow dives was 116 ± 43 s, which was not different from the duration of the deep dives (120 ± 18 s, NS; Fig. 2). The mean duration of recovery periods between subsequent dives was 539 ± 258 s.

Temperature, measured by the logger on the back inside the wetsuit, was the same at the surface before diving, at 31.5 °C (NS). The temperature dropped by 0.5 ± 0.2 °C during shallow dives, and by 1.5 ± 0.6 °C during deep dives (*P* < 0.001), reflecting the colder water temperature at depth. For the pooled data, there was a correlation between temperature drop across dives and diving depth (*r* = 0.676, *P* < 0.01).

None of the divers suffered from blackout or showed any symptoms of loss of motion control or pulmonary barotrauma after resurfacing. Responses were highly individual, as seen in Fig. 3, but average responses are reported to enable comparison of shallow and deep dives.

### Arterial oxygen saturation

**Fig. 4:**
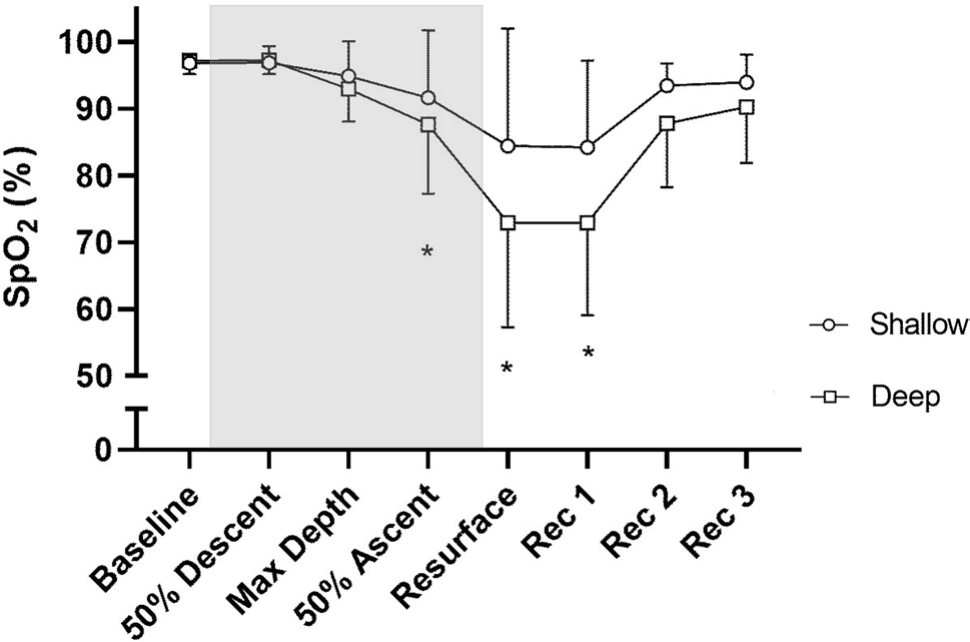
Mean ± SD oxygen saturation (SpO_2_) for shallow and deep dives at different dive points. Shaded area indicates the dive period. Rec = Recovery. * = Significant difference between shallow dives and deep dives (*P* ≤ 0.05). Baseline period is an average of 60 s prior to diving; dive points are average values of 10 s bins surrounding those points concerning dive depth; recovery periods are average values of 10 s bins, starting 5 s following resurfacing

Mean baseline SpO_2_ before diving was 97 ± 2% in both shallow and deep dives (NS; Fig. 4). Mean SpO_2_ remained similar during the first half of both types of dives, even at maximal depth (95 ± 5% in shallow and 93 ± 5% in deep dives; *P* = 0.140; Fig. 4).

Similar SpO_2_ desaturation patterns were also observed during the second half of the dives in shallow and deep dives, although the level of desaturation increased with depth. Halfway through the ascent, SpO_2_ had been reduced to 92 ± 10% in shallow dives compared to 88 ± 10% in deep dives (*P* = 0.048; Fig. 4). Upon resurfacing, SpO_2_ was 84 ± 18% in shallow dives compared to 73 ± 16% in deep dives (*P* = 0.023).

Mean SpO_2_ during 5–15 s after surfacing was still reduced at 84 ± 13% after shallow dives and 73 ± 14% after deep dives (*P* = 0.044). During the period 15–25 s after surfacing, it had increased to 93 ± 3% in shallow and 88 ± 10% in deep dives (*P* = 0.059), and during 25–35 s into recovery it was 94 ± 4% for shallow and 90 ± 8% for deep dives (*P* = 0.179; Fig. 4).

Mean SpO_2min_ was 74 ± 17% after shallow dives and 58 ± 17% after deep dives (P = 0.029).

**Fig. 5:**
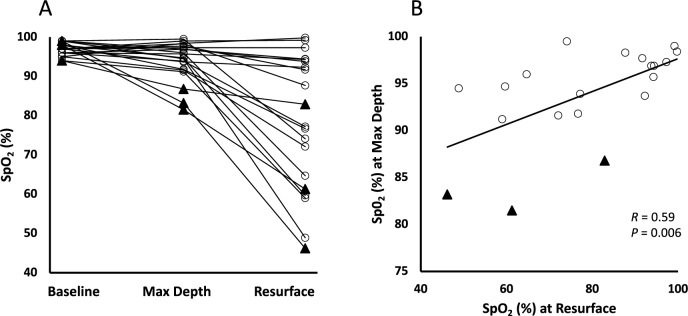
**A** Individual SpO_2_ measurements at Baseline, the individual maximal diving depth reached (Max Depth) and Resurface. **B** Correlation plot of arterial oxygen saturation (SpO_2_) at Max Depth plotted against SpO_2_ at Resurface. Closed triangles indicate dives with early oxygen desaturation at maximal depth, open circles indicate all other dives

Three divers developed hypoxia early, with SpO_2_ already below 90% at maximal depth, and two of these developed profound hypoxia (SpO_2_ ≤ 65%) upon resurfacing (46% and 61%; Fig. 5a). Four additional divers who remained above 90% SpO_2_ at depth developed profound hypoxia upon resurfacing (Fig. 5a). For all divers pooled, there was a positive correlation between SpO_2_ at maximal depth and upon resurfacing (*r* = 0.59, *P* = 0.006; Fig. 5b).

### Heart rate

**Fig. 6:**
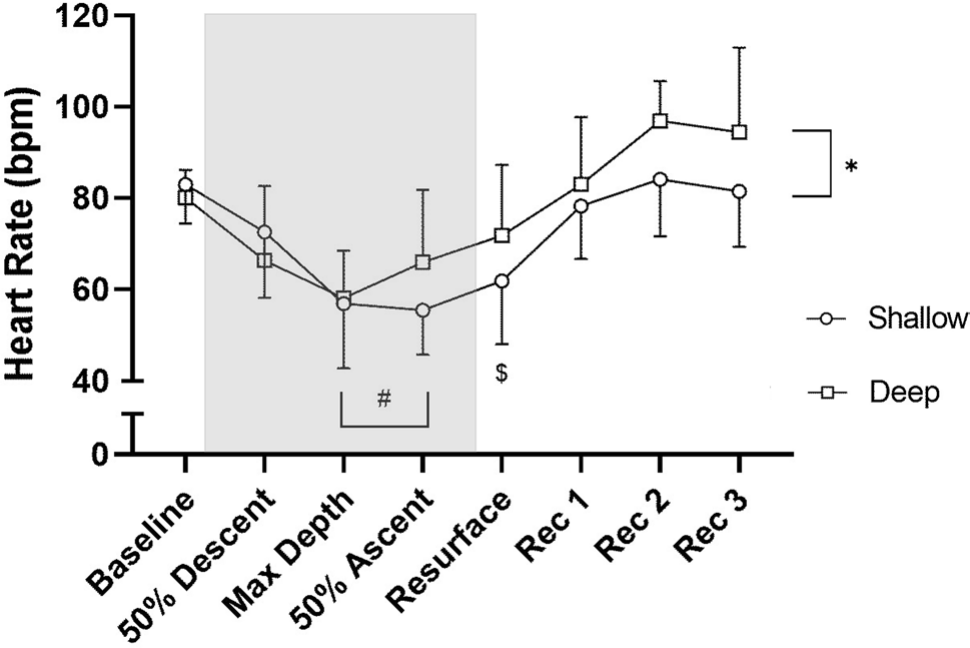
Mean ± SD heart rate (HR) for shallow and deep dives at different dive points. Shaded area indicates the dive period. Rec = Recovery. * = Significant difference between shallow dives and deep dives (*P* ≤ 0.05); # = Different compared to Baseline, Recovery 1, Recovery 2 and Recovery 3 (*P* ≤ 0.05); $ = Different compared to Baseline, Recovery 2 and Recovery 3 (*P* ≤ 0.05). Baseline period is an average of 60 s prior to diving; dive points are average values of 10 s bins surrounding those points concerning dive depth; recovery periods are average values of 10 s bins, starting 5 s following resurfacing

Mean baseline HR before diving was similar for shallow (83 ± 9 bpm) and deep dives (80 ± 6 bpm; NS; Fig. 6). Shallow and deep dives resulted in similar mean HR response patterns, with pronounced bradycardia at maximal depth (57 ± 14 bpm and 58 ± 10 bpm, respectively; *P* = 0.001 compared to baseline; Fig. 6).

**Fig. 7:**
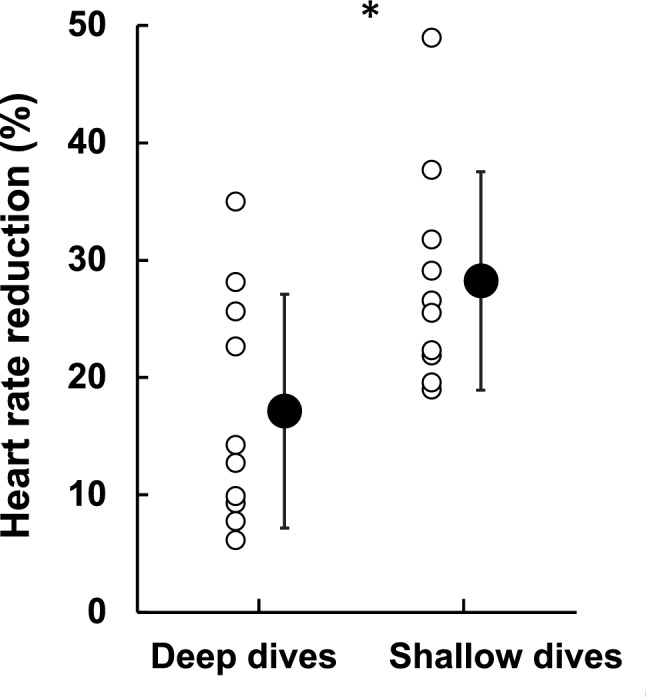
Heart rate (HR) reduction for deep dives and shallow dives, calculated as the difference between baseline HR and the diving HR. * indicates *P* = 0.005

Mean HR during shallow dives was 59 ± 9 bpm, which was lower than the 66 ± 9 bpm in deep dives (*P* = 0.002). HR was thus reduced more from baseline in shallow dives (28.2 ± 9.3%) than in deep dives (17.1 ± 10.0%; *P* = 0.005; Fig. 7). HR_min_ was not different between shallow (39 ± 5 bpm) and deep dives (39 ± 9 bpm; NS), however. HR was highly variable across the dives, increasingly so in deep dives (Fig. 3). HR_min_ tended to develop later in shallow dives (on average at 63% of dive duration) than in deep dives ( at 42% of dive duration; *P* = 0.088).

HR remained below baseline levels during ascent, during both shallow and deep dives (55 ± 10 bpm and 66 ± 16 bpm, respectively; *P* = 0.001 compared to baseline), and also upon resurfacing (62 ± 14 bpm and 72 ± 16 bpm, respectively; *P* = 0.05 compared to baseline; Fig. 6). It had returned to baseline levels during the first recovery period. Averaged across all dive points, HR was 5 ± 2 bpm lower in shallow dives than in deep dives (*P* = 0.011). The lowest individual HR observed during diving was 28 bpm during a shallow dive, corresponding to 67% HR reduction from baseline. HR_min_ occurred at different parts of the dive for different individuals (Fig. 3).

## Discussion

The freedivers developed greater oxygen desaturation when surfacing from deep dives than from shallow dives, despite similar dive duration. This reinforces the classic belief that “shallow water blackout” arises from the rapid drop in alveolar oxygen pressure on ascent, which may reduce or even reverse oxygen uptake (Lanphier and Rahn 1963). However, our underwater recordings of HR and SpO_2_ have allowed identification of several other likely causal mechanisms of increased oxygen desaturation and subsequent blackout risk in deep diving.

One important factor which would contribute to increased desaturation was the higher overall HR in deep dives compared to shallow dives, which likely reflects a higher level of exertion during deep dives. The lower buoyancy with greater depth due to the lung- and wetsuit compression required higher exertion levels to ascend compared to shallower depths, which is evident from the increase in HR—especially after the turn in deep but not in shallow dives. We found that HR during deep dives was 7 ± 5 bpm higher than during shallow dives, which could have contributed to the larger decrease in SpO_2_ during deep dives. It has previously been shown in laboratory experiments that apneic exercise will increase HR and rate of oxygen desaturation (Wein et al. 2007), but comparisons of detailed events during deep and shallow dives of similar duration in the field have not been analyzed previously. With the higher overall HR reflecting higher metabolic rate, it may be assumed that carbon dioxide- and lactate accumulation was higher during deep dives, leading to a lower pH. This causes a right shift of the oxyhemoglobin dissociation curve, reducing the oxygen affinity of hemoglobin and thereby promoting the unloading of oxygen to peripheral tissue (West and Luks, 2021). This contributes to desaturation, compromising brain oxygenation.

The relatively high HR in deep dives shows that the cardiovascular diving response, typically resulting from apnea and facial cold stimulation (Schuitema and Holm 1988), is compromised during deep dives. An intermediate response seems to result when the parasympathetically induced bradycardia is counteracted by the sympathetically induced exercise tachycardia. This attenuation of the diving response in deep dives occurred despite a greater drop in diver´s skin temperature, reflecting exposure to lower water temperatures at depth. Reduction in skin temperature is a greater determinant of the magnitude of bradycardia than absolute temperature (Schagatay and Holm 1996). Ferrigno et al. (1997) showed in a wet hyperbaric chamber study that diving bradycardia was increased in colder water, but our results indicate that other factors are more important in determining HR. It is also evident that the lung compression at depth does not enhance the diving response in real dives, in contrast with previous laboratory-based results that demonstrated more pronounced bradycardia with smaller lung volumes during apnea (Andersson and Schagatay 1998b). This contradiction illustrates the importance of studying the combined effects of several factors in real diving to reveal their net effect and physiological consequences.

The parasympathetically induced bradycardia, combined with sympathetically induced exercise tachycardia, may result in an “autonomic conflict” (Shattock and Tipton 2012; Costalat et al. 2021), which is supported by the highly variable HR we observed. The observation of an irregular HR, especially during deep dives, may support the theory that arrhythmias arising as the result of an autonomic conflict can potentially cause blackout (Mulder et al. 2023). An arrhythmic HR pattern was observed in one diver during a maximal static apnea, which resulted in a blackout despite similar dive duration and oxygen saturation as other divers in the same competition (Mulder et al. 2023). Compared to shallow dives, an increased autonomic conflict in deep dives, arising from the greater exertion-related sympathetic stimulus versus the greater temperature- and lung compression-induced parasympathetic drive, is likelier. Hypoxia may also enhance bradycardia (Costalat et al. 2015) but despite augmented hypoxia by the end of deep dives, the HR increased.

The pressure at maximal depth of deep dives in our study, on average 6 atm, would have compressed lungs to below residual volume. Increased alveolar oxygen pressure promotes oxygen uptake, assuming lung function is intact. However, we observed that despite the expected hyperoxia at depth, three divers showed desaturation, indicating that lung compression with depth may, at least in some individuals, reduce oxygen uptake. This supports earlier observations using arterial blood gas sampling (Bosco et al. 2018). While the effect may be most evident in the individuals showing early desaturation, we speculate that partial atelectasis or sub-symptomatic pulmonary edema may also have occurred in the other four divers that desaturated below 65% after dives. These divers are apparently at greater risk of developing blackout after deep dives, and underwater pulse oximetry could provide a tool to identify them.

The resurfacing oxygen saturation values are similar to previous work (Mcknight et al. 2021; Mulder et al. 2021a) or lower (Bosco et al. 2018; Barković et al. 2023). The observed levels of 73% on average after deep dives, with several divers reaching levels below 65%, demonstrates a significant risk for hypoxic blackout during planned training dives. The deep dives performed in this study represented ~ 75% of the divers’ personal best and could, therefore, be considered more strenuous on an individual level than in previous studies of sled-assisted dives to 40 m (Bosco et al. 2018) or dives to < 60% of personal best (Barković et al. 2023).

The observed HR results contrast with previous findings from static apnea at varying depths. Marabotti et al. (2009) showed that cardiac output decreased more during static apnea at 5 m depth compared to static apnea at the surface, due to both decreased HR and stroke volume. However, in agreement with our study, Marongiu et al. (2015) showed an increase in HR, stroke volume and cardiac output during ascent from dives to 20 and 30 m depth compared to resting at the surface.

The diving HR profiles obtained were different than previous laboratory-based findings that observed lowest HR towards the end of the apnea breaking point in trained divers (Costalat et al. 2015). This likely arises from chemoreflex and/or baroreflex activation, as both hypoxia and increased blood pressure are expected at this stage (Tibes and Stegemann 1969; Taboni et al. 2019). In the current study, individual HR_min_ varied within the same individual as well as between conditions. HR_min_ was more often found toward the end of the shallow dives, while in deep dives it more often occurred in the first half of the dive. This could possibly be explained by the longer “free-fall phase” of the deeper dives, where bradycardia is not compromised by exertion (Schagatay 2011; Mulder et al. 2021a), in combination with the increased physical exertion during the ascent phase, compared to shallow dives.

During deep dives in the sea, Lemaître et al. (2013) showed that elite divers reached a stable HR following approximately 20 s after submersion and HR remained below baseline during the entire dive. In the current study, HR was significantly lower than baseline at each of these points but increased during ascent, indicating that diving bradycardia may not suppress exercise-induced tachycardia in such situations.

Sea lions’ diving bradycardia is enhanced at depth (McDonald and Ponganis 2014), but the current study’s results suggest that the same does not hold true for humans. That the diving response does not fully develop despite lung compression and cold-water exposure at depth—factors found in the laboratory to individually cause stronger bradycardia (Schagatay and Holm 1996; Andersson and Schagatay 1998b)—is remarkable and shows the importance of studying these factors in combination during field studies with continuous direct measurement. The findings of this study further demonstrate that trained freedivers are frequently exposed to severe hypoxia even during submaximal deep diving, confirming earlier studies that they are more tolerant to hypoxia than non-divers (Elia et al. 2021). While training was conducted without blackout occurring, the margin for error is small at such oxygen saturation levels, further illustrating the need for in-water safety divers even during non-competition freediving.

This study also shows that underwater continuous pulse oximetry may facilitate detection of early desaturation in individuals at risk for developing severe hypoxia. This data could potentially be displayed in a subject-borne device, as a warning or guidance tool. It is also a considerably simpler method for conducting research, particularly in comparison to underwater blood sampling.

### Study limitations

Pulse oximetry is a practical and accurate tool for continuous measurement of SpO_2_ and HR, but accuracy may decrease at lower saturation and perfusion values (Chan et al. 2013). The underwater pulse oximeter employed in the current study has been evaluated against arterial blood gas sampling and showed good agreement across the saturation levels reported here (unpublished observations). We did not control for depth and duration of dives a priori. This may reduce the comparability of results somewhat but is also ethically defensible from a risk perspective. Mean responses are presented when comparing the effects of depth, but a general limitation is the large individual variation, and it should be further studied how consistent responses are within individuals. The study population was trained freedivers and findings are, therefore, limited to this group; previous research has demonstrated differences in responses between trained and untrained divers (Schagatay and Holm 1996; Elia et al. 2021).

## Conclusions

Deep diving increases risk for hypoxic blackout, as demonstrated by more pronounced arterial oxygen desaturation following deep dives compared to shallow dives of similar duration. This elevated risk is likely caused by a combination of factors: (1) a more rapid drop in alveolar pressure during ascent from deep dives, as indicated by the greater drop in SpO_2_ during this phase; (2) increased oxygen consumption due to increased exertion during ascent from deep dives, as indicated by higher HR during deep dives; (3) a compromised diving response in deep dives, despite colder temperatures and smaller lung volumes due to increased lung compression at depth, shown by the higher HR during deep dives; (4) increased “autonomic conflict” in deep dives between the parasympathetically induced diving response and the sympathetically induced exercise tachycardia as indicated by highly variable HR, possibly augmenting arrhythmia prevalence and potentially increasing risk of blackout; and (5) increased lung compression during deep dives that may, at least in some individuals, reduce oxygen uptake, possibly caused by partial atelactasis or sub-symptomatic pulmonary edema. Underwater continuous pulse oximetry may be a useful tool in both identifying risk levels and providing warning signals to ameliorate such risk. To determine the respective influence of the different causal factors identified requires further study.

## Data Availability

The datasets generated during the current study are not publicly available as this conflicts with our ethics agreement with the participants. Requests to obtain confidential access to the datasets should be directed to the corresponding author.

## Abbreviations

ANOVA: Analysis of variance
atm: Atmosphere
bpm: Beats per minute
HR: Heart rate
HR_min_: Lowest HR attained
msw: Meters of sea water
NS: Non-significant
SD: Standard deviation
S_p_O_2_: Arterial oxygen saturation measured by pulse oximetry
S_p_O_2min_: Lowest SpO_2_ attained
